# Blood and Tissue Enzymatic Activities of GDH and LDH, Index of Glutathione, and Oxidative Stress among Breast Cancer Patients Attending Referral Hospitals of Addis Ababa, Ethiopia: Hospital-Based Comparative Cross-Sectional Study

**DOI:** 10.1155/2018/6039453

**Published:** 2018-03-26

**Authors:** Mohammed Mehdi, M. K. C. Menon, Nebiyou Seyoum, Mahteme Bekele, Wondimagegn Tigeneh, Daniel Seifu

**Affiliations:** ^1^Department of Biochemistry, College of Health Sciences, School of Medicine, Addis Ababa University, Addis Ababa, Ethiopia; ^2^Department of Surgery, College of Health Sciences, School of Medicine, Addis Ababa University, Addis Ababa, Ethiopia; ^3^Department of Surgery, St. Paul Hospital, Millennium Medical College, Addis Ababa, Ethiopia; ^4^Department of Oncology, College of Health Sciences, School of Medicine, Addis Ababa University, Addis Ababa, Ethiopia

## Abstract

The exact cause of breast cancer is unknown; it is a multifactorial disease. It is the most diagnosed and the second killer cancer among women. Breast cancer can be originated from tissues of breast or secondary from other organs via metastasis. Generally, cancer cells show aberrant metabolism and oxidative stress when compared to noncancerous tissues of breast cancer patients. The current study aims at evaluating glutamate and glucose metabolism through GDH and LDH enzyme activities, oxidant, and antioxidative status among breast cancer patients attending referral hospitals of Addis Ababa, Ethiopia. *Result*. Catalytic activities of glutamate dehydrogenase, lactate dehydrogenase, and oxidative stress index were significantly increased in both serum (4.2 mU/ml, 78.6 mU/ml, and 3.3 : 1, resp.) and cancerous tissues (1.4 mU/ml, 111.7 mU/ml, and 2.15 : 1, resp.) of breast cancer patients as compared to those in serum of control group (3.15 mU/ml, 30.4 mU/ml, and 2.05 : 1, resp.) and noncancerous tissues of breast cancer patients (0.92 mU/ml, 70.5 mU/ml, and 1.1 : 1, resp.) (*P* ≤ 0.05). Correspondingly, ratios of reduced to oxidized glutathione were significantly decreased in both serum (20 : 1) and cancerous tissues (23.5 : 1) of breast cancer patients when compared to those in serum of control group (104.5 : 1) and noncancerous tissues of breast cancer patients (70.9 : 1) (*P* ≤ 0.05). *Conclusion*. Catalytic activities of GDH and LDH, ratios of GSH to GSSG, and concentration of TOS among breast cancer patients were significantly higher than were those among control group and noncancerous tissues of breast cancer patients, while TAC of breast cancer patients is significantly lower than that of control group and normal tissues of breast cancer patients.

## 1. Background

Breast cancer is a multifactorial and devastating disease. It is characterized by its uncontrolled growth and spread of atypical breast cells [1, 2]. Globally among women, breast cancer is the most frequently diagnosed and second leading cause of cancer death. As the global burden of breast cancer and its comorbidities has increased, it is evident that novel diagnostic and therapeutic approaches will be necessary to address the breast cancer epidemic. According to the report of American Cancer Society, from 2009 to 2013 in the USA, the incident rate of breast cancer was 123.3/100,000 and death rate from 2010 to 2014 was 21.2/100,000. In 2017, it is estimated to diagnose new 252,710 invasive and 63,410 in situ cases and 40,610 death of breast cancer patients. Considering incidence trend of breast cancer from 2004 to 2013, invasive breast cancer seems to be stable in white women, but in black women, it was increased by 0.5% [3].

Otto Warburg and his coworkers were the first to study the metabolism of cancer in the 1920s. According to their thought, cancer is a metabolic disease. When normal cells are deprived 35% of their oxygen supply, they will either die or turn into cancer cells. Cancer cells are not like a normal cell, lack the “intelligence” as a result of their division and will be uncontrolled. Such uncontrolled oncogene-driven proliferation of cancer cells and the absence of an efficient vascular bed cause low oxygen tension (hypoxia), forced cancer cells to live in conditions of aerobic glycolysis. Under aerobic conditions, tumor tissues can metabolize approximately tenfold more glucose to lactate in a given time than can normal tissues. Such acidic condition favors tumor invasion and suppresses anticancer immune effectors. Lactate that is produced by tumor cells can be taken up by stromal cells, to regenerate pyruvate that either can be squeezed out to refuel the cancer cell or can be used for oxidative phosphorylation [4–8].

Additional to the Warburg effect, intermediates of glycolysis in cancer cells can be used for the synthesis of protein, nucleotides, and fatty acids [9–11]. Shunting of glucose into aerobic glycolysis causes a reduction in Krebs cycle intermediates “pulls” glutamate through GDH generating *α*-ketoglutarate (*α*KG). This is proved by the higher steady state of NH_4_^+^/Gln ratio greater than 1. Ammonium to alanine produced ratio will be increased. This will indicate the increased GDH and decreased ALT flux results in reduced intramitochondrial pyruvate (metabolized in the cytosol to lactate). Thus, the increased glutamate flux through GDH generates *α*KG while sparing ketoacid consumption (reduced transamination) [12].

Sparing of glutamate for Krebs cycle intermediates may cause oxidative stress in cancer patients; this may be due to the decreased synthesis of glutathione (major internal antioxidant). All in all, oxidative stress occurs as a result of an imbalance or state of oxidation exceeds the antioxidant systems of the body [13]. Reducing substances in the human body controls the status of over oxidation, and a continuing imbalance in support of oxidation causes different problems when it beats the limit of such control. Free radicals and antioxidant can reinforce differing impacts on cells according to their concentration. Reactive oxygen species may participate in carcinogenesis through induction of gene mutations that result in cell damage and the consequences of signal transduction and transcription factors, and the redox status of cancer cells usually differs from that of normal cells [14]. Because of metabolic and signaling aberrations, cancer cells exhibit elevated ROS levels and it is balanced by an increased antioxidant capacity, which suggests that high ROS levels may constitute a barrier to tumorigenesis [15].

The present study aimed at identifying early metabolic biomarkers for diagnosis, prognosis, and therapeutic targets for breast cancer diseases. This might be possible through evaluation of GDH and LDH enzyme activities, reduced and oxidized glutathione, and oxidative stress index (TOS/TAC) of cancerous tissues and serum of breast cancer patients as compared to serum of control group and adjacent noncancerous tissues of the same patients attending referral hospitals of Addis Ababa, Ethiopia.

### 1.1. Methodology

A comparative cross-sectional study was conducted at five major referral hospitals [Tikur Anbessa Specialized Hospital, Zewditu Memorial Hospital, St. Paul Specialized Hospital, Menelik the Second Hospital, and Yekatit 12 Hospital] and one health center of Addis Ababa, Ethiopia. The study was conducted from July 2015 to May 2017; accordingly, 27 breast cancer patients and 27 normal individuals as control group were included.

### 1.2. Blood and Tissue Sample Collection Procedure

Before surgery, the patients were well informed and made aware of the aim of the study. Then, blood samples and their responses to questionnaires were collected by professional nurses. After surgery, the cancerous tissue and adjacent noncancerous tissue samples were collected from the patients.

### 1.3. Tissue Samples

**Figure 1:**
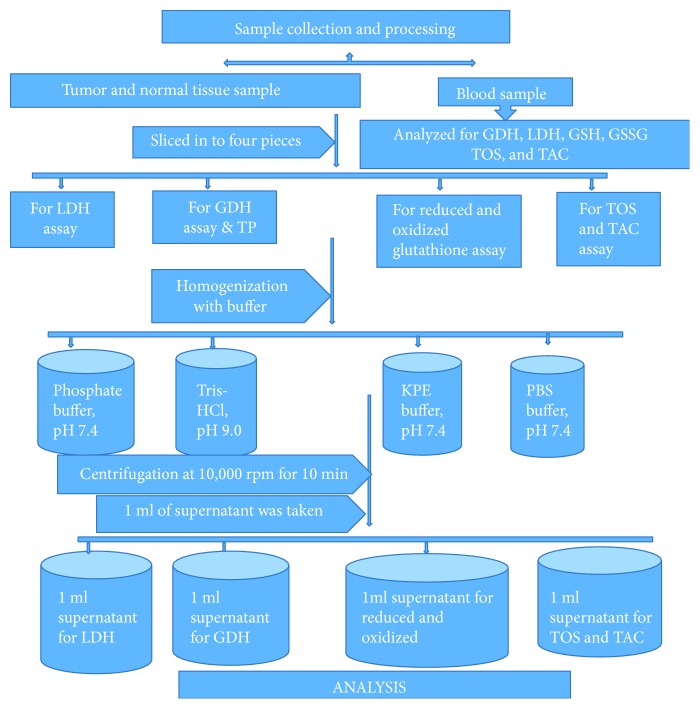
Chart showing the workflow of sample processing, Addis Ababa, Ethiopia, 2015–2017.

A total of 54 tissue samples (27 tumor tissues and 27 normal tissues) were collected from 27 breast cancer patients. The collection and fixation of cancerous tissue and adjacent noncancerous tissue was done by attending surgeons of the Surgery Department at the operation room using cold phosphate buffer saline (PBS) and dimethyl sulfoxide (DMSO) and then stored at −80°C of the deep freezer until analysis. During the actual process, portions of the solid tumor, free of fat, connective tissue, necrotic debris, and blood, were cut into pieces of approximately 3 × 3 × 3 mm and frozen quickly (Figure 1). The staging of breast cancer was done by pathologist, and it was based on tumor size, nodal involvement, and metastasis (TNM) staging method.

### 1.4. Blood Samples

A total of 54 blood samples (27 breast cancer patients and 27 control group) with a volume of 3–5 ml were collected using serum separator tube (SST). Serum samples were harvested into Eppendorf tube after the blood samples were centrifuged at 4000 rpm for five minutes and stored at −80°C deep freezer until the laboratory analysis.

### 1.5. Homogenization of Tissue Sample

Tissue samples were thawed and sliced into five, approximately 50 to 100 mg wet weight. Each aliquot was homogenized in cold 0.05 M KPE buffer (pH 7.4), 0.1 M phosphate buffer (pH 7.4), 0.1 M Tris-HCl buffer (pH 9.0), and 0.1 M phosphate buffer saline (pH 7.4) for the analysis of GSH and GSSG, LDH, GDH, TOS, and TAC, respectively. Then, the supernatant of the homogenates was taken for determinations of GSH and GSSG, LDH, GDH, TOS, and TAC after centrifugation in 10,000 rpm for 10 min (Figure 1).

### 1.6. Chemicals and Equipment Used

Glutamate dehydrogenase (GDH), lactate dehydrogenase (LDH), reduced form glutathione (GSH), oxidized form glutathione (GSSG), total protein, total oxidative status (TOS), and total antioxidant capacity (TAC) were determined using the following kits and chemicals bought from Sigma-Aldrich, Merck, and BDH Chemical Company: potassium dihydrogen orthophosphate (KH_2_PO_4_), dipotassium hydrogen orthophosphate (K_2_HPO_4_), EDTA sodium salt, sulfosalicylic acid, Triton X-100, DTNB, *β*-NADPH, glutathione reductase, reduced form glutathione (GSH), glutathione (disulfide form) (GSSG), triethanolamine and 2-vinylpyridine, xylenol orange, bovine serum albumin (BSA) (Sigma-Aldrich), BCA protein assay reagents, sodium chloride (NaCl), sulfuric acid (H_2_SO_4_), glycerol, ferrous ammonium sulfate, *o*-dianisidine dihydrochloride, sodium acetate (CH_3_COONa), glacial acetic acid, 35%, H_2_O_2_ solution, 2,2′-azino-bis(3-ethylbenzthiazoline-6-sulfonic acid) (ABTS), and Trolox (6-hydroxy-2,5,7,8-tetramethylchroman-2-carboxylic acid). The equipment used was Eppendorf tube and Falcon centrifuge tube (Multifuge), ultraviolet/visible spectrophotometer (Jenway, 6705, UK), ELISA plate reader (Biotest, 2001, Austria), homogenizer (Heidolph, RZR 2100), water path (GFL, 1002, Germany), and magnetic stirrer.

### 1.7. Glutamate Dehydrogenase (GDH) Assay Principle

The change of NAD^+^ to NADH is measured spectrophotometrically at 340 nm and is relative to the activity of glutamate dehydrogenase [16]. The test results were expressed as mU/l.

(1) $$\begin{array}{c} Glutamate+{NAD}^{+}\overset{GDH}{⇄}\;\alpha ‐Ketoglutaric\;acid+NADH. \end{array}$$

### 1.8. Lactate Dehydrogenase (LDH) Assay Principle

It was determined based on the principles of Vassault. The consumption of NADH was measured using spectrophotometer at 340 nm [17].

(2) $$\begin{array}{c} NADH+H^{+}+Pyruvate\overset{LDH}{⇄}Lactate+{NAD}^{+}. \end{array}$$

### 1.9. Total Glutathione (GSH) Assay Principle

It was determined based on the principles of Rahman et al. [18]. The assay depends on the reaction of GSH with DTNB that produces the TNB chromophore and oxidized glutathione–TNB adduct (GS–TNB). The rate of formation of TNB, measured at 412 nm, is proportional to the concentration of GSH in the sample. The disulfide product (GS–TNB) is then reduced by GR in the presence of NADPH, recycling GSH back into the reaction. Because GR reduces the GSSG formed into 2GSH, the amount of glutathione measured represents the sum of reduced and oxidized glutathione in the sample ([GSH] total = [GSH] + 2 × [GSSG]) [18].

(3) $$\begin{array}{c} 2GSH+DTNB\overset{GR}{\to }TNB+GS‐TNB,\\ GS‐TNB+NADPH\overset{GR}{\to }2GSH+{NADP}^{+}. \end{array}$$

### 1.10. Oxidized Glutathione (GSSG) Assay Principle

It was determined based on the principles of Rahman et al. [18]. The principle used GSSG reductase recycling method. By monitoring NADPH spectrophotometrically at a wavelength of 340 nm, the amount of GSSG was determined. The samples are treated with 2-vinylpyridine, which covalently reacts with GSH (but not GSSG). The excess 2-vinylpyridine is neutralized with triethanolamine [18].

(4) $$\begin{array}{c} GSSG+NADPH\overset{GR}{\to }2GSH+{NADP}^{+}. \end{array}$$

### 1.11. Total Protein Assay Method and Principle

Determination of total protein in serum and tissue of the study participants was done based on the method of Smith et al. [19]. The principle of this method is that proteins in the sample reduce Cu^+2^ to Cu^+1^ in an alkaline solution (the biuret reaction) and result in a purple color formation by bicinchoninic acid (BCA). The absorbance was read at wavelength of 562 nm.

### 1.12. Total Oxidant Status (TOS) Assay Principle

It was determined based on the principles of Erel [20]. In this process, oxidants present in the sample oxidize the ferrous particle *o*-dianisidine complex to the ferric particle. The oxidation reaction is upgraded by glycerol molecules, which are richly present in the reaction medium. A colored compound is formed when the ferric ion reacts with xylenol orange in an acidic medium. The color strength, which can be measured spectrophotometrically at 560 nm wavelength, is correlated to the total quantity of oxidant molecules present in the plasma. The assay is aligned with hydrogen peroxide, and the outcomes are expressed as far as *μ*molar hydrogen peroxide equivalent per liter (*μ*mol H_2_O_2_ Eq/l) [20].

### 1.13. Total Antioxidant Capacity (TAC) Assay Principle

It was determined based on the principles of Koracevic et al. [21]. In this technique, the hydroxyl radical, the most powerful natural radical, is generated by the Fenton reaction and it responds with the colorless substrate *o*-dianisidine to create the dianisyl radical, which is splendid yellowish brown in color. Upon the addition of sample, the oxidative responses started by the hydroxyl radicals present in the reaction are scavenged by the antioxidant agents present in the sample, keeping the color change and consequently giving a viable estimation of TAC [21]. The test results were expressed as mmol Trolox Eq/l.

### 1.14. Determination of Oxidative Stress Index (OSI)

It was calculated based on the method of Erel [20]. The proportion of TOS to TAC is acknowledged as the oxidative stress index (OSI). For estimation, the subsequent unit of TAC is changed over to mmol/l, and the OSI value is computed [20].

(5) $$\begin{array}{c} OSI\;\left(subjective\;unit\right)=\frac{TOS\left(\mu mol\;H_{2}O\right)}{TAC\left(mmol\;TroloxEq/l\right)}\times 100. \end{array}$$

### 1.15. Data Processing and Software Used in Statistical Analysis

All data were checked, cleared and fed into EpiData (version 3.5.1, 2008), and then exported to SPSS (version 22.0, 2012, America) software for statistical analysis. Descriptive analysis, Spearman correlation, linear regression, independent sample *t*-test, and one-way ANOVA followed by post hoc analysis were used for this study. All data were expressed in mean ± SD, and *P* ≤ 0.05 was considered as statistically significant.

### 1.16. Ethical Approval

The study was ethically approved from the Ethical Review Committee of Biochemistry Department College of Health Sciences, Addis Ababa University, with protocol number 09/15 and meeting number DRERC 09/15.

## 2. Results

### 2.1. Socio-Demographic Profile

A total of 54 (27 breast cancer patients and 27 normal individuals as control group) participants were recruited. These were from five major referral hospitals of Addis Ababa, Ethiopia, and one health center. These were Tikur Anbessa Specialized Hospital (TASH), St. Paul Specialized Hospital (SPH), Zewditu Memorial Hospital (ZMH), Yekatit 12 Hospital (YH), Menelik the Second Hospital (MH), and Teklehaimanot Health Center (THC). Most of the participants were from Tikur Anbessa Specialized Hospital (13 patients, 48.1%) and St. Paul Specialized Hospital (6 patients, 22.2%).

Socio-demographic profiles of participants are presented in Table 1. All participants were female, and their mean age was 44.93 with a minimum age of 25 to a maximum age of 68. Thirteen of them were less than or equal to 40 years old, and fourteen of them were greater than 40 years old. Consecutively, sex- and age-matched control samples were also collected.

Out of the 27 breast cancer patients, 17 (63.0%) were living in urban areas and 10 (37.0%) were living in rural areas. Twelve of breast cancer patients (44.5%) were illiterate, 17 (63.0%) of them were married, 15 (55.6%) of them gave birth, had at least 1 and at most 4 children, 15 (55.6%) of them feed their children with breast milk, 14 (51.9%) of them used birth control, 16 (59.3%) of them were premenopausal, and 3 (11.1%) of them were obese (Table 1).

### 2.2. Clinical and Histopathological Findings

Clinical and histopathological results of all breast cancer patients were studied and tabulated in Table 2. From each breast cancer patient, tumor tissue, noncancerous tissue (5 cm away from cancerous tissue), and blood sample were collected. Histology of tumor tissues was graded as low-grade or well-differentiated (9 patients, 33.3%), intermediate grade (intermediately differentiated) (10 patients, 37.0%), and high-grade (poorly differentiated) (8 patients, 29.6%) cases. Staging of tumor tissues was done based on tumor size; all tumor tissues were classified into five stages. Out of which, 5 patients (18.5%) were in stage zero, 4 patients (14.8%) were in stage one, 7 patients (25.9%) were in stage two, 8 patients (29.6%) were in stage three, and 3 patients (11.1%) were in stage four. Based on invasiveness, tumor tissues were categorized into invasive ductal carcinoma (11 patients, 40.7%), invasive lobular carcinoma (5 patients, 18.5%), ductal carcinoma in situ (8 patients, 29.6%), and lobular carcinoma in situ (3 patients, 11.1%) (Table 2).

### 2.3. Biochemical Analysis

#### 2.3.1. Serum and Tissue Enzymatic Activity of Glutamate Dehydrogenase (GDH)

Glutamate dehydrogenase (GDH) activity was determined and normalized by dividing with total protein in serum and tissues of the study participants. The catalytic activities of GDH in serum samples of breast cancer patients and control group were significantly different (*P* < 0.05) (95% CI (0.8–1.3)). Glutamate dehydrogenase in serum samples of breast cancer patients were 4.20 ± 0.72 mU/l, whereas in control group, it was 3.15 ± 0.69 mU/l. Similarly, catalytic activities of GDH in cancerous and noncancerous tissues of breast cancer patients were assessed and were significantly different (*P* < 0.05) (95% CI (0.12–0.82)). The cancerous tissues had enzymatic activities of GDH in comparison with noncancerous tissues (0.92 ± 0.73 and 1.4 ± 0.88 mU/l, resp.) (Tables 3 and 4).

### 2.4. Serum and Tissue Enzymatic Activities of Lactate Dehydrogenase (LDH)

Enzymatic activities of LDH in serum and tissue samples from breast cancer patients were investigated in comparison to those in serum samples from control group and normal tissues of breast cancer patients. Results were normalized by the amount of total protein in serum and tissue of the study participants. Serum LDH activities of breast cancer patients were significantly higher than were those of control group (78.6 ± 113 and 30.4 ± 32.6 mU/l, resp.) (*P* < 0.05) (95% CI (3.4–92.9)) (Table 4). Similarly, cancerous tissues had a higher LDH activities than had noncancerous tissues (111.7 ± 23.2 and 70.5 ± 10.7 mU/l), and it was statistically significant (*P* < 0.05) (95% CI (−7.5–89.9)) (Tables 3 and 4).

### 2.5. Serum and Tissue Levels of Glutathione

The concentration of reduced and oxidized glutathione in serum and tissue samples of breast cancer patients was examined (Tables 3 and 4). Results were normalized by the amount of total protein in serum and tissue of the study participants. Oxidized glutathione in serum of breast cancer patients was significantly (*P* ≤ 0.05) higher than was that of control group (0.51 ± 0.2 and 0.2 ± 0.1 *μ*M/*μ*g of total protein, resp.). Similarly, cancerous tissues of breast cancer patients showed a significantly (*P* ≤ 0.05) higher oxidized glutathione than did noncancerous tissues of breast cancer patients (0.47 ± 0.3 and 0.21 ± 0.1 *μ*M/*μ*g of total protein, resp.).

**Figure 2:**
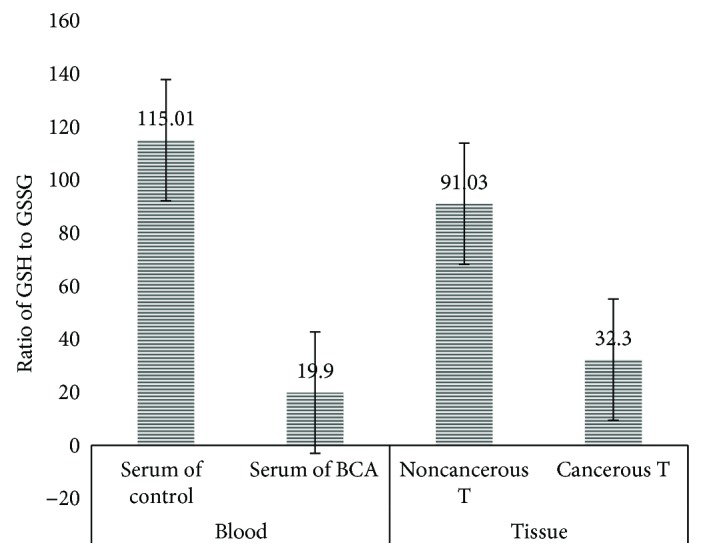
A bar graph showing glutathione index in serum and cancerous tissues of breast cancer (BCA) patients in comparison to serum of control group and noncancerous tissues of breast cancer patients, Addis Ababa, Ethiopia, 2015–2017.

Consecutively, serum of breast cancer patients was significantly lower in reduced glutathione as compared to serum of control group (10.2 ± 2.9 and 20.9 ± 2.6 *μ*M/*μ*g of total protein, resp.) (*P* < 0.05). Correspondingly, reduced glutathione in cancerous tissue and noncancerous tissue of breast cancer patients was statistically different (*P* ≤ 0.05); tumor tissues had a lower reduced glutathione than had normal tissues (11.03 ± 2.0 and 14.9 ± 2.7 *μ*M/*μ*g of total protein, resp.). Furthermore, ratios of reduced (GSH) to oxidized glutathione (GSSG) in serum and cancerous tissues of breast cancer patients (19.9 : 1 and 32.3 : 1, resp.) were significantly (*P* ≤ 0.05) decreased as compared to those in serum samples of control group and noncancerous tissue of breast cancer patients (30.7 : 1 and 75 : 1, resp.) (Figure 2).

### 2.6. Serum and Tissue Levels of TOS and TAC

The concentration of total oxidative status (TOS) of breast cancer patients and control group was examined (refer to Tables 4 and 5). Total oxidative status in serum of breast cancer patients (3.3 ± 1.7 *μ*mol H_2_O_2_ Eq/l) was significantly (*P* ≤ 0.05) higher than was that of control group (2.3 ± 2.0 *μ*mol H_2_O_2_ Eq/l) (Tables 3 and 4). Within tissue samples of breast cancer patients, total oxidative status in tumor tissues (2.15 ± 1.8 *μ*mol H_2_O_2_ Eq/l) was significantly (*P* ≤ 0.05) higher than was that in normal tissues (1.1 ± 0.5 *μ*mol H_2_O_2_ Eq/l) (Tables 3 and 4).

In serum sample of breast cancer patients, there was a significantly (*P* ≤ 0.05) lower amount of TAC concentration (83.5 ± 30.3 mmol Trolox Eq/l) than that of control group (100.9 ± 29.8 mmol Trolox Eq/l). In tissue sample of breast cancer patients, TAC in tumor tissues was significantly (*P* ≤ 0.05) lower in the concentration of TAC (161.6 ± 50.8 mmol Trolox Eq/l) than that in normal tissues (188.9 ± 26.7 mmol Trolox Eq/l) and their mean difference among tissue samples of breast cancer patients was statistically significant (Tables 3 and 4).

### 2.7. Oxidative Stress Index (OSI)

**Figure 3:**
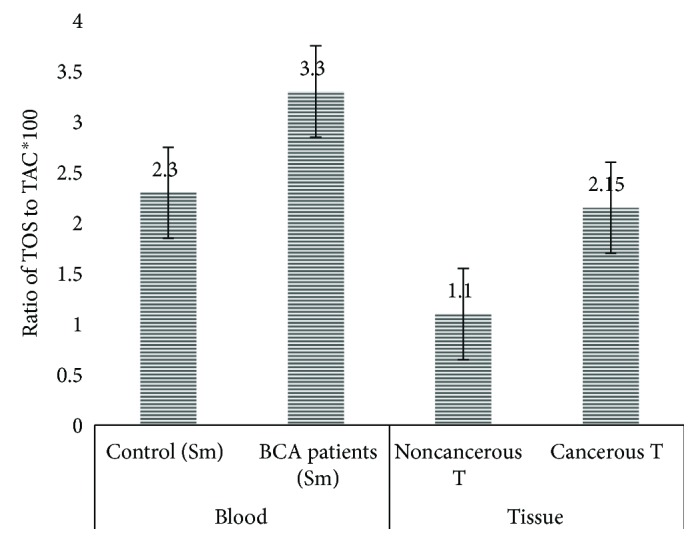
A bar graph showing oxidative stress index in serum and cancerous tissues of breast cancer (BCA) patients in comparison to serum of control group and noncancerous tissue of breast cancer patients, Addis Ababa, Ethiopia, 2015–2017.

Likewise, OSI in serum and tissue samples of breast cancer patients and control group was explored. Serum samples of breast cancer patients had a significantly higher OSI value (3.3 ± 1.7) than control group (2.3 ± 2.0), and the difference was statistically significant (*P* = 0.006). Within tissues of breast cancer patients, cancerous tissues had a higher OSI (2.15 ± 1.8) value than noncancerous tissues (1.1 ± 0.5) (*P* = 0.002) (Tables 3 and 4 and Figure 3).

### 2.8. Comparison of Different Parameters of Serum and Tissues within the Numerous Stages Identified in Breast Cancer Patients

Blood and tissue parameters of GDH, LDH, GSH, GSSG, TOS, TAC, and OSI were compared with the stages of breast cancer patients (refer to Table 5). Even though it was not statistically significant (*P* > 0.05), serum glutamate dehydrogenase enzyme activity was higher in stage zero (4.6 ± 0.4 mU/l) and lower in stage four (3.7 ± 0.45 mU/l), whereas tissue glutamate dehydrogenase enzyme activities were higher in stage three (1.9 ± 1.1 mU/l) and lower in stage zero (0.7 ± 0.2 mU/l) and it was statistically significant (*P* ≤ 0.05).

The catalytic activities of serum lactate dehydrogenase were higher in stage four (341.8 ± 41.4 mU/l) and lower in stage one (42.4 ± 4.1 mU/l), and the mean difference of stage four with stages zero, one, two, and three was statistically significant (*P* ≤ 0.05) (Table 5). The catalytic activities of tissue lactate dehydrogenase were higher in stage two (138.7 ± 61.9 mU/l) and lower in stage zero (77.7 ± 27.1 mU/l); however, their mean difference was not statistically significant (*P* > 0.05).

Serum and tissue levels of total antioxidant capacity were lower in stages zero and four, whereas in stages two and three, there was a higher value but it was not statistically significant (*P* > 0.05). Similarly, serum and tissue levels of total oxidative status were not consistent and fluctuate among the stages of breast cancer patients. The mean difference of total oxidative status in serum of stage zero of breast cancer patients was significantly different in comparison to the other stages one, three, and four (*P* = 0.003, 0.002, and 0.045, resp.) (Table 5).

**Figure 4:**
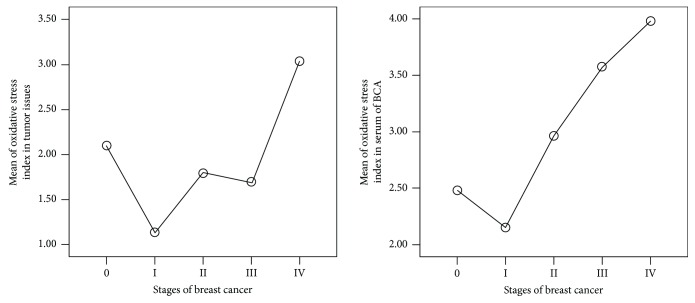
Mean plots of oxidative stress index in serum and tumor tissue samples of breast cancer patients, Addis Ababa, Ethiopia, 2015–2017.

The oxidative stress index of breast cancer patients was analyzed and correlated with stage of cancer (Figure 4). There was significantly higher oxidative stress in stage four of breast cancer patients than in the other stages (*P* < 0.05), whereas it was lower in stage one.

## 3. Discussion

Currently, the prevalence of cancer has grown into a major public anxiety, as it is becoming the major cause of morbidity and mortality worldwide. More than 60% of cancer cases occur in Africa, Asia, Central, and South America. According to the International Agency for Research on Cancer [22], about 715,000 new cancer cases and 542,000 cancer deaths occurred in 2008 in Africa. In Ethiopia, there is no country-wide cancer registry; however, based on Addis Ababa cancer registry, a total of 5701 cancer cases were registered from September 2011 to August 2014. Among those 3820 (67%) were females and 1881 (33%) were males. The most common type of cancers among females were cancers of the breast (33%), cervix (17%), and ovary (6%), while among male cancers of colorectal (19%), leukemia (18%), and prostate (11%) [23]. Hence, an early biomarker for diagnosis, prognosis, and a potential treatment target for breast cancer is required.

In the present study, serum and tissue levels of glutamate dehydrogenase (GDH), lactate dehydrogenase (LDH), reduced glutathione (GSH), oxidized glutathione (GSSG), total oxidative status (TOS), and total antioxidant capacity (TAC) were determined in search of a potential biomarker for diagnosis, prognosis, and treatment target for breast cancer disease. Those serum and tissue parameters were studied on 54 (27 breast cancer patients and 27 age- and sex-matched apparently healthy control group) participants.

As current study revealed, significantly higher enzymatic activities of GDH and LDH, ratios of TOS to TAC (OSI), and lower ratios of GSH to GSSG in serum and tissue samples from breast cancer patients were observed as compared to noncancerous tissue of the same patients and serum samples of control group.

Activities of GDH were significantly (*P* = 0.011) increased (almost 1.5 times) in both serum and tumor tissues of breast cancer patients as compared to adjacent noncancerous tissues of the same patient or serum samples of control group. Furthermore, between stages of breast cancer, stage zero has the lowest and stage three has the highest activity of GDH in tumor tissues of breast cancer patients and the mean difference is statistically significant (*P* = 0.029). These findings agreed with the studies of Koppenol et al. [10], Toyokuni et al. [24], Koukourakis et al. [25], Lu et al. [26], Liu et al. [27], and Agrawal et al. [28]. The possible reason for high catalytic activities of GDH in cancer cells may be due to the fact that either it is important for redox homeostasis in cancer cells [29] or overexpression of GDH promoted cell proliferation, migration, and invasion *in vitro*, whereas loss of function of GDH had the opposite effect [27].

Previous works suggest that GDH enzymes are important in cancer cell either for synthesizing Krebs cycle intermediates (*α*-ketoglutarate and subsequent metabolite fumarate) or for synthesizing protein and fatty acid from citrate which originate from *α*-ketoglutarate. In addition to that, the substrate of GDH, glutamate itself is a substrate for antioxidant (GSH) and nucleotide synthesis in the cancer cell. These metabolic changes support the production of intermediates for cell growth and division and are regulated by both oncogenes and tumor suppressor genes, in a number of key cancer-producing pathways [30, 31].

Similarly, the catalytic activities of LDH were significantly increased in both cancerous tissue and serum of breast cancer patients when compared to noncancerous tissues of breast cancer patients and serum of control group. The results of current study showed that mean values of LDH were significantly (*P* < 0.05) lower in noncancerous tissues (70.5 ± 10.7) mU/l than in tumor tissues (111.7 ± 23.2 mU/l) of breast cancer patients. Patients with a higher clinical stage had higher LDH activity than lower stages, and there is a significant difference of mean between stage four (341.8 ± 41.4) and stage zero (42.4 ± 4.1 mU/l), stage one (63.8 ± 5.1 mU/l), stage two (67.7 ± 3.1 mU/l), and stage three (131.3 ± 8.4 mU/l) (*P* < 0.05). These observations were in agreement with the studies of Agrawal et al. [28] and Talaiezadeh et al. [32].

The high activities of LDH in cancer cells may be due to the process of high cell proliferation, migration, or invasion than normal cells. That is to say, large cancer cell population requires a higher and rapid energy source as compared to normal cell population. In order to meet this large and rapid energy demand, cancer cells use LDH activity as one option which is helpful for metabolic requirements and aerobic glycolysis of malignant cells. The possible mechanisms of high LDH activities in the cancer cell may be due to higher expressions of LDH gene in cancer cells as compared to normal cells [28, 32].

Generally, tissues have different rates of metabolic activity and oxygen consumption. When cells have a high production of reactive oxygen species than cellular antioxidant defenses, attempts by the cells to remove these toxic species induce oxidative stress. Oxidative stress has long been implicated in cancer development and progression [33]. The current study examined reduced glutathione (GSH), oxidized glutathione (GSSG), total oxidative status (TOS), and total antioxidant capacity (TAC) of serum and cancerous tissue of breast cancer patients in comparison to serum of control group and noncancerous tissue of breast cancer patients as a tool for assessing oxidative stress. The current study's result showed that concentration of oxidized glutathione in both serum and tissue samples from breast cancer patients was significantly increased as compared to serum of control group and noncancerous tissues of breast cancer patients (*P* < 0.05). Cancerous tissues (0.47 ± 0.3 *μ*M/*μ*g of total protein) have a higher mean value than noncancerous tissues (0.21 ± 0.1 *μ*M/*μ*g of total protein). Whereas, the counterpart, concentration of reduced glutathione in both serum and cancerous tissue was significantly decreased when compared to serum of control group and noncancerous tissues of breast cancer patients (*P* < 0.05), tumor tissues (11.03 ± 2.0 *μ*M/*μ*g of total protein) have lower mean value than normal tissues (14.9 ± 2.7 *μ*M/*μ*g of total protein).

In the same way, ratios of reduced (GSH) to oxidized glutathione (GSSG) in serum and cancerous tissues of breast cancer patients (19.9 : 1 and 32.3 : 1, resp.) were decreased as compared to those in serum samples of control group and noncancerous tissues of breast cancer patients (30.7 : 1 and 75 : 1, resp.) (Figure 4). These observations agreed with the report of Perry et al. [34] and Gamcsik et al. [35]. Perry and his colleagues [34] reported that GSSG levels in primary breast tumors were more than twice the levels found in normal breast tissue and levels in lymph node metastases were more than four times the levels found in normal breast tissue. Another group Gamcsik and his colleagues [35] reported that oxidized glutathione levels in breast tumors are higher than in disease-free breast tissue.

The possible justification for these results may be due to unusual levels of oxidative stress in breast cancer as oxidized glutathione in healthy tissue normally is below 5% of the reduced from. This might be due to the content of GSH in some tumor cells that is typically associated with higher levels of GSH-related enzymes, such as *γ*-glutamylcysteine ligase (GCL) and *γ*-glutamyltranspeptidase (GGT) activities, as well as a higher expression of GSH-transporting export pumps [34, 35]. Barranco and his colleagues [36] reported that the larger ratios of tumor glutathione to normal tissue glutathione, the poorer prognosis of cancer and less survival.

Moreover, the concentration of total oxidative status (TOS) and total antioxidant capacity (TAC) and their ratios (OSI) in breast cancer patients and control group were also investigated. The results revealed that TOS was significantly elevated in both serum and tumor tissues of breast cancer patients than serum of control group and noncancerous tissues of breast cancer patients (2.6 ± 1.1 *μ*mol H_2_O_2_ Eq/l in the serum of breast cancer patients and 1.8 ± 1.0 *μ*mol H_2_O_2_ Eq/l in serum control group) (*P* = 0.001). Similarly, it was 2.8 ± 1.1 *μ*mol H_2_O_2_ Eq/l in tumor tissues of breast cancer patients and 2.0 ± 0.9 *μ*mol H_2_O_2_ Eq/l in normal tissues of the same breast cancer patients (*P* ≤ 0.001).

Correspondingly, breast cancer patients have a significantly lower concentration of total antioxidant capacity (TAC) in both serum (0.83 ± 0.3 mmol Trolox Eq/l) and tumor tissue (1.61 ± 0.5 mmol Trolox Eq/l) than serum samples of control group (1.09 ± 0.3 mmol Trolox Eq/l) and in adjacent normal tissues (1.88 ± 0.26 mmol Trolox Eq/l) of breast cancer patients (*P* < 0.05).

These findings agreed with the study of others finding [37–40]. Erten Şener and his colleagues [37] found that TAC was 2.01 ± 0.01 mmol Trolox Eq/l in patients with breast cancer and 2.07 ± 0.03 mmol Trolox Eq/l in control group (*P* < 0.05), and Zowczak-Drabarczyk and his colleagues [38] found that TAC in breast cancer patients was 1.35 mmol Trolox Eq/l and in control group was 1.61 mmol Trolox Eq/l (*P* < 0.05). The findings of TAC were also in lined with the study of former whom found a significantly higher value of oxidative status, as compared to control group [39, 40]. Furthermore, oxidative status in other types of cancer patients such as thyroid cancer and colorectal cancer patients reported increased concentration of TOS [37, 38, 41, 42].

Consistently, ratios of TOS to TAC (OSI) in serum and tissue samples from breast cancer patients were significantly different as compared to those in serum samples from control group and noncancerous tissue of the same breast cancer patients. Serum samples of breast cancer patients have had a significantly higher ratio of total oxidative status to total antioxidant capacity (OSI) value (3.3 ± 1.7) than control group (2.3 ± 1.5) (*P* = 0.006). Likewise, tumor tissues of breast cancer patients had significantly higher value of OSI (2.15 ± 1.8) than noncancerous tissue (1.1 ± 0.5) (*P* = 0.002). This finding agreed with the study of Feng and his colleagues. They found a significantly higher values of OSI in breast cancer patients when compared to control group [43].

Surprisingly, even within the different stages of breast cancer patients, OSI values were different. Lower stages (0 and one) have lower values of OSI than the higher stages (two to four) of breast cancer patients. Mean difference of OSI between stages one and four in tumor tissue of breast cancer patients was significantly (*P* = 0.037) different. These results are supported by the study of Zarrini and his colleagues [44]. They reported that patients in advanced stages had lower serum antioxidant capacity and higher lipid peroxidation levels than control group [44].

The possible reason for high oxidative stress in breast cancer cells may be due to oxygen radical production by the macrophages. In addition, tumor necrosis factor-*α* is secreted by tumor-associated macrophages and is known to induce cellular oxidative stress. Determination of oxidative stress in cancer cells is useful either to detect the increase of the mutation rate during accelerated tumor progression or to activate growth-promoting signaling pathways. It is also helpful to adapt oxidative stress which results in increased resistance to therapy or to increase blood supply to tumor cells. It was also useful in evaluating the risk of invasion and metastasis of cancerous cells [45, 46].

Reczek and Chandel explained the dual role of reactive oxygen species (ROS) in cancer. ROS can promote protumorigenic signaling, facilitating cancer cell proliferation, survival, and adaptation to hypoxia. Conversely, ROS can promote antitumorigenic signaling and trigger oxidative stress-induced cancer cell death [47].

Furthermore, oxygen radicals are powerful DNA damaging agents, either ROS causes strand breaks or alterations in guanine and thymine bases or sister chromatid exchanges. This may inactivate additional tumor suppressor genes within tumor cells or further increase expression of proto-oncogenes. Genetic instability due to persistent carcinoma cell oxidative stress will, therefore, increase the malignant potential of the tumor [45].

## 4. Conclusion

Enzymatic activities of glutamate dehydrogenase and lactate dehydrogenase, the concentration of reduced and oxidized glutathione, and the ratio of total oxidative status and total antioxidant capacity (OSI) in serum and tumor tissues of breast cancer patients were determined. In conclusion, enzymatic activities of glutamate dehydrogenase and lactate dehydrogenase as well as ratios of total oxidative status to total antioxidant capacity were significantly increased in serum and tumor tissues of breast cancer patients as compared to serum of control group and noncancerous tissues of breast cancer. However, ratios of reduced to oxidized glutathione were significantly decreased in both serum and cancerous tissues of patients as compared to serum of control group and noncancerous tissues of the same breast cancer patients. Furthermore, marital status, bearing a child, and breast feeding have a lower risk for breast cancer than unmarried women who never bore a child and who did not breast feed, whereas birth control has a higher risk for breast cancer than nonuser women. Therefore, glutamate dehydrogenase, lactate dehydrogenase, and oxidative stress play a critical role in breast cancer progression and may be an ideal therapeutic target for regulation of breast cancer disease.

## Figures and Tables

**Table 1:** Descriptive analysis of socio-demographic profile of breast cancer (BCA) patients and control group at the five referral hospitals and one health center of Addis Ababa, Ethiopia, 2015–2017.

Socio-demographic data of BCA and control group		BCA patients (*N* = 27) *N* (%)	Control group (*N* = 27) *N* (%)
Age (yr.)	≤40	13 (48.1)	14 (51.9)
	>40	14 (51.9)	13 (48.1)

Residence	Urban	17 (63.0)	19 (70.4)
	Rural	10 (37.0)	8 (29.6)

Education level	Illiterate	12 (44.5)	0 (0.0)
	High school or less	11 (40.7)	14 (51.9)
	College and above	4 (14.8)	13 (48.1)

Marital status	Single	7 (25.9)	18 (66.7)
	Married	17 (63.0)	9 (33.3)
	Widowed	3 (11.1)	0 (0.0)

Child birth	Yes	15 (55.6)	7 (25.9)
	No	12 (44.4)	20 (74.1)

No. of children	0	12 (44.4)	20 (74.1)
	1–4	14 (51.9)	7 (25.9)
	≥5	1 (3.7)	0 (0.0)

Breast feeding	Yes	15 (55.6)	7 (25.9)
	No	12 (44.4)	20 (74.1)

Birth control	Yes	14 (51.9)	5 (18.5)
	No	13 (48.1)	22 (81.5)

Menopausal status	Pre	16 (59.3)	21 (77.8)
	Post	11 (40.7)	6 (22.2)

Body mass index (kg/m^2^)	Under weight (<18.5)	4 (14.8)	2 (7.4)
	Normal weight (18.5–24.9)	14 (51.9)	18 (66.7)
	Over weight (25–29.9)	6 (22.2)	4 (14.8)
	Obese (≥30)	3 (11.1)	3 (11.1)

**Table 2:** Descriptive analysis of clinical and pathological profiles of breast cancer (BCA) patients attending referral hospitals of Addis Ababa, Ethiopia, 2015–2017.

Clinicopathological profile of BCA (*N* = 27)		*N* (%)
Family history of BCA	Yes	7 (25.9)
	No	20 (74.1)

Location of breast cancer	Right breast	17 (63)
	Left breast	10 (37)

Tumor size	pT1 (0.1–2 cm)	9 (33.3)
	pT2 (2–5 cm)	8 (29.6)
	pT3 (>5 cm)	3 (11.1)
	pT4 (extension to the chest wall/skin)	7 (25.9)

Nodal status	pN0	10 (37.0)
	pN1	12 (44.4)
	pN2	3 (11.1)
	pN3	2 (7.4)

Metastasis	Mx	2 (7.4)
	M0	23 (85.2)
	M1	2 (7.4)

Stage of BCA	0	5 (18.5)
	I	4 (14.8)
	II	7 (25.9)
	III	8 (29.6)
	IV	3 (11.1)

Grading	Low grade (well differentiated)	9 (33.3)
	Intermediate grade (moderately–differentiated)	10 (37.1)
	High grade (poorly–differentiated)	8 (29.6)

Histology of cancer	Invasive ductal carcinoma	11 (40.7)
	Invasive lobular carcinoma	5 (18.5)
	Ductal carcinoma in situ	8 (29.6)
	Lobular carcinoma in situ	3 (11.1)

**Table 3:** Comparative mean analysis of serum enzymatic activities of GDH and LDH, concentration of glutathione, and the oxidative stress index of breast cancer (BCA) patients (*N* = 27) and control group (*N* = 27), Addis Ababa, Ethiopia, 2015–2017.

Serum parameters	Control group	BCA patients	Mean diff.	*P* value	95% CI
GDH (mU/l)	3.15 ± 0.69	4.20 ± 0.72	1.04	≤0.001	(0.8–1.3)
LDH (mU/l)	30.4 ± 32.6	78.6 ± 113	48.2	0.036^∗^	(3.4–92.9)
GSH (*μ*M per *μ*g of protein)	20.9 ± 2.6	10.2 ± 2.9	−10.7	≤0.001	(4.3–6.0)
GSSG (*μ*M per *μ*g of protein	0.2 ± 0.1	0.51 ± 0.2	0.31	≤0.001	(0.8–1.2)
TOS (*μ*mol H_2_O_2_ Eq/l)	2.32 ± 1.0	2.75 ± 1.1	0.43	≤0.001	(0.39–1.27)
TAC (mmol Trolox Eq/l)	100.9 ± 29.8	83.5 ± 30.3	−13.65	0.017^∗^	(−24.7 to −2.6)
OSI (ratio of TOS/TAC^∗^100)	2.3 ± 1.5	3.3 ± 1.7	1.0	0.006^∗^	(0.32–1.67)
Total protein (*μ*g/ml)	59.7 ± 29.3	208 ± 11.8	148.22	0.001^∗^	(143.56–152.87)

^∗^The mean difference is significant at *P* value ≤ 0.05.

**Table 4:** Comparative mean analysis of tissue enzymatic activities of GDH and LDH, concentration of glutathione, and the oxidative stress index of noncancerous tissues (*N* = 27) and cancerous tissues (*N* = 27), Addis Ababa, Ethiopia, 2015–2017.

Tissue parameters	Normal tissue	Tumor tissue	Mean diff.	*P* value	95% CI
GDH (mU/l)	0.92 ± 0.73	1.40 ± 0.88	0.5	0.011^∗^	(0.12–0.82)
LDH (mU/l)	70.5 ± 10.7	111.7 ± 23.2	41.2	0.009^∗^	(−7.5 to 89.9)
GSH (*μ*M per *μ*g of protein)	14.9 ± 2.7	11.03 ± 2.0	−3.87	0.029^∗^	(−0.2 to 0.8)
GSSG (*μ*M per *μ*g of protein)	0.21 ± 0.1	0.47 ± 0.3	0.26	0.003^∗^	(0.09–0.02)
TOS (*μ*mol H_2_O_2_ Eq/l)	2.1 ± 0.9	3.5 ± 1.1	1.4	≤0.001	(0.33–1.22)
TAC (mmol Trolox Eq/l)	188.9 ± 26.7	161.6 ± 50.8	−27.32	0.01^∗^	(−47.42 to −7.2)
OSI (ratio of TOS/TAC^∗^100)	1.1 ± 0.5	2.15 ± 1.8	1.05	0.002^∗^	(0.3–1.22)
Total protein (*μ*g/ml)	149.4 ± 54.2	194.9 ± 27.4	45.5	0.001^∗^	(34.7–56.4)

^∗^The mean difference is significant at *P* value ≤ 0.05.

**Table 5:** A one-way ANOVA (post hoc) analysis of serum and tissue parameters in control subjects and pathologically confirmed breast cancer patients participated from five hospitals of Addis Ababa, Ethiopia (*N* = 27), 2015–2017.

Serum and tissue parameters of BCA	Sample	Stages of breast cancer patients				
		Stage 0 (*N* = 5)	Stage I (*N* = 4)	Stage II (*N* = 7)	Stage III (*N* = 8)	Stage IV (*N* = 3)
GDH	S	4.6 ± 0.4	4.1 ± 0.5	4.13 ± 0.91	4.2 ± 0.9	3.7 ± 0.45
	T	0.7 ± 0.2^a^	1.2 ± 0.6	1.24 ± 0.71	1.9 ± 1.1^a^	1.78 ± 1.15
LDH	S	77.7 ± 27.1	80.9 ± 38.7	138.7 ± 61.9	57.6 ± 5.4	90.0 ± 11.7
	T	42.4 ± 4.1^b^	63.8 ± 5.1^b^	67.7 ± 3.1^b^	131.3 ± 8.4^b^	341.8 ± 41.4^b^
GSH	S	5.4 ± 2.4	5.9 ± 0.5	5.8 ± 1.8	7.0 ± 3.3	6.3 ± 0.6
	T	1.0 ± 0.3	1.2 ± 0.5	2.0 ± 1.9	1.4 ± 1.5	1.5 ± 0.7
GSSG	S	1.1 ± 0.5	1.2 ± 0.1	1.1 ± 0.4	1.4 ± 0.6	1.2 ± 0.1
	T	0.3 ± 0.1	0.3 ± 0.04	0.2 ± 0.1	0.3 ± 0.1	0.2 ± 0.03
TOS	S	1.4 ± 0.4^c^	3.4 ± 1.3^c^	2.4 ± 0.7	3.2 ± 1.16^c^	2.8 ± 0.6^c^
	T	3.2 ± 0.3	2.3 ± 0.4	3.3 ± 1.6	2.5 ± 1.2	2.4 ± 0.8
TAC	S	0.90 ± 0.29	0.84 ± 0.14	0.87 ± 0.21	0.90 ± 0.34	0.77 ± 0.47
	T	1.84 ± 0.23	1.44 ± 0.33	1.86 ± 0.40	1.46 ± 0.59	1.12 ± 0.83
OSI	S	2.47 ± 0.87	2.15 ± 1.29	2.96 ± 1.8	3.57 ± 2.06	3.98 ± 1.23
	T	2.1 ± 0.87	1.13 ± 0.84^d^	1.78 ± 0.4	1.69 ± 0.83	3.03 ± 2.9^d^
Total protein	S	212.1 ± 14.4	209.7 ± 8.6	197.3 ± 8.6	210.9 ± 11.6	215.3 ± 4.34
	T	175.9 ± 51.9	193.8 ± 30.7	195.1 ± 6.5	200.8 ± 12.8	212.2 ± 27.3

^a^Mean difference of GDH between stages 0 and III of tissue sample (*P* ≤ 0.05), ^b^mean difference of LDH between stages IV and 0, I, II, and III of tissue sample (*P* ≤ 0.001, 0.001, 0.001, and ≤0.05, resp.), ^c^mean difference between stages 0 and I, III, and IV (*P* ≤ 0.05) of blood sample, and ^d^mean difference of OSI among stages I and IV in tissue of BCA (*P* ≤ 0.05) were statistically significant. NB: measuring units of GDH and LDH are in mU/l, GSH and GSSG were in *μ*M/*μ*g of total protein, TOS is in *μ*mol H_2_O_2_ Eq/l, TAC is in mmol Trolox Eq/l, and total protein is in *μ*g/ml.

## Abbreviations

GDH: Glutamate dehydrogenase
GSH: Reduced glutathione
GSSG: Oxidized glutathione
LDH: Lactate dehydrogenase
TAC: Total antioxidant capacity
TOS: Total oxidative stress.
